# Photo Quiz: Brown, thick-walled cells with evident septation in a purulent sample from a mason

**DOI:** 10.1128/jcm.01552-25

**Published:** 2026-04-23

**Authors:** Xiujiao Xia, Zehu Liu

**Affiliations:** 1 Department of Dermatology, Hangzhou Third People’s Hospital, Hangzhou Third Hospital Affiliated to Zhejiang Chinese Medical University, Hangzhou, China; Mayo Clinic Minnesota, Rochester, Minnesota, USA

## PHOTO QUIZ

**Fig 1:**
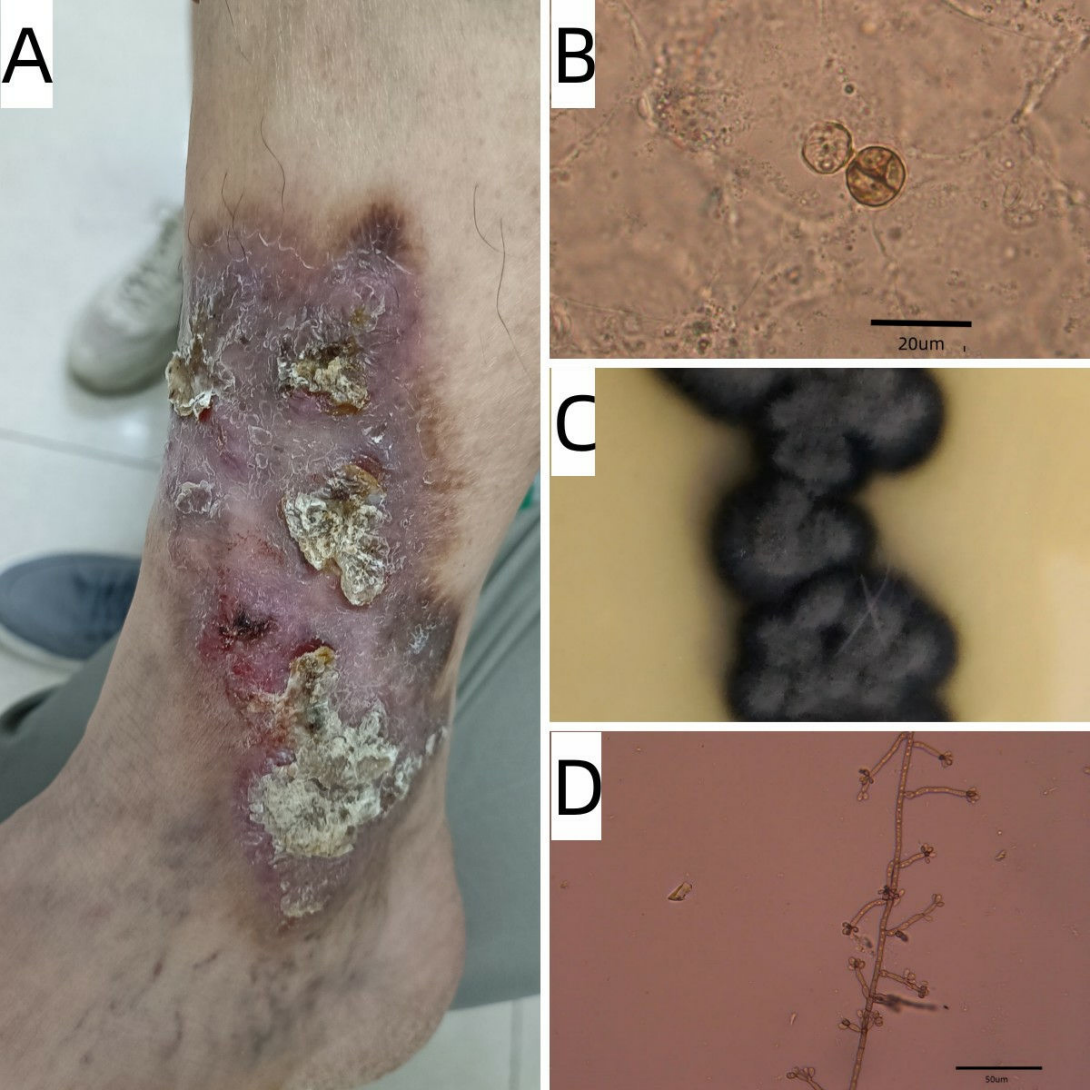
An extensive, irregular, dark-red plaque covered with grayish-white crusts on the right lower extremity (**A**). Clusters of spherical, septate, brown, thick-walled fungal cells (B, KOH, ×1,000). *F. monophora* colonies on Sabouraud dextrose agar at 25°C on day 13 (**C**). Slide culture of *F. monophora* on potato dextrose agar at 25°C on day 10 (D, ×400).

A 55-year-old otherwise healthy male mason from Zhejiang Province presented to our dermatology clinic with a 14-year history of progressively increasing plaques over the right lower extremity. The patient reported that the skin lesion developed after a local car accident, during which he sustained a right ankle injury. The resulting wound healed poorly and gradually evolved into papules and nodules that enlarged to form a crusted plaque. Despite seeking care at multiple hospitals, the skin lesion failed to improve. Dermatological examination revealed an extensive, irregular, dark-red plaque covered with grayish-white crusts on the right lower extremity, near the medial malleolus (Fig. 1A); no other body areas were involved. Compression of the lesion with a sterile lancet resulted in the expression of purulent material. Microscopic examination with 10% potassium hydroxide (KOH) of the purulent specimen showed spherical cells occurring in clusters, which were brown, septate, and thick walled (Fig. 1B).

What is your diagnosis?

## ANSWER TO PHOTO QUIZ

The patient was diagnosed with chromoblastomycosis (CBM). CBM is an uncommon subcutaneous fungal disease caused by dematiaceous fungi, prevalent in tropical and subtropical regions (1). Several dematiaceous fungi are recognized causative agents of CBM. The most prevalent genera are *Fonsecaea* and *Cladophialophora*, while *Exophiala*, *Phialophora*, and *Rhinocladiella* are also occasionally implicated. These fungi are typically saprobes found in soil and plant debris. Infection usually occurs via traumatic inoculation of fungal elements through the skin, most commonly on the extremities (2). Following a 13-day incubation on Sabouraud dextrose agar at 25°C, fungal colonies from the exudate were slow growing, black, and smut-like (Fig. 1C and D). The isolate was definitively identified as *Fonsecaea monophora* (100% identity) by internal transcribed spacer sequencing, and the sequence was deposited in GenBank (accession no. PX426321). The patient was started on a regimen of oral itraconazole 400 mg daily and remains on this treatment.

The confirmation of CBM is contingent upon the identification of muriform cells (sclerotic bodies) in infected tissues (3), which is a mandatory diagnostic criterion. Microscopically, these cells are spherical to polyhedral (chestnut-like), range from 5 to 12 μm in diameter, and possess thick, dark-pigmented walls. Their defining feature is the presence of both transverse and longitudinal septa, resulting in a distinctive brick-walled morphology. They can be found either singly or in clusters (4).

Melanized fungi are polymorphic microorganisms that exhibit significant morphological diversity owing to their plasticity and adaptability to diverse organic and inorganic environments. Following transcutaneous implantation, propagules of CBM agents display remarkable cellular and morphological plasticity. During infection, cellular differentiation adopts a meristematic pattern, characterized by isodiametric swelling and cross-septation, leading to the formation of so-called muriform cells (5). These muriform cells are regarded as an evolutionary adaptation that facilitates survival within the host microenvironment (6). They are directly associated with an intense granulomatous inflammatory response and contribute to immune evasion, thereby promoting the establishment of chronic disease. Dematiaceous fungi are also etiological agents of phaeohyphomycosis (PHM). PHM occurs predominantly in immunocompromised individuals and is histopathologically characterized by the presence of melanized septate hyphae within tissues, occasionally accompanied by yeast-like cells. The innate immune response to these fungal elements can induce tissue necrosis, which may progress to destructive lesions (7). Melanin in the cell wall serves as a significant virulence factor in these dematiaceous fungi. During infection, melanin interferes with the production of nitric oxide and suppresses phagocytosis (8).

Direct mycological examination represents a rapid, straightforward, and cost-effective approach for diagnosing CBM, particularly in resource-limited settings. Clinicians and microbiologists should be aware that the detection of muriform cells in skin scrapings or biopsy specimens enables a rapid diagnosis and the prompt initiation of appropriate therapy (9). For melanized fungi, special staining is generally unnecessary, as it may mask their inherent pigmentation. It is essential that during specimen collection, efforts are made to obtain pus or tissue scrapings from areas containing black dots, which typically correspond to fungal elements (10).

CBM progresses slowly, and the initial lesions with mild manifestations are usually asymptomatic and do not interfere with daily activities. Patients often delay seeking treatment for years or even decades; therefore, CBM is notoriously challenging to treat and often refractory to multiple therapeutic modalities. Reported management strategies include antifungal agents, immunomodulatory therapy, physical interventions, photodynamic therapy, and surgical excision. Among these, itraconazole is the first-line antifungal of choice (2), and a treatment course of at least 6 months is typically required to achieve a favorable clinical response (11).
